# Investigation of glucose-6-phosphate dehydrogenase (G6PD) deficiency prevalence in a *Plasmodium vivax*-endemic area in the Republic of Korea (ROK)

**DOI:** 10.1186/s12936-020-03393-4

**Published:** 2020-09-01

**Authors:** Wonsig Lee, Sang-Eun Lee, Min Jun Lee, Kyung Tae Noh

**Affiliations:** 1 Department of Infectious Disease Research, Armed Forces Medical Research Institute, 90bun, Jaunro, Yuseong-gu, Daejeon, 34059 Republic of Korea; 2 Division of Vectors & Parasitic Diseases, Center for Laboratory Control of Infectious Diseases, Korea Centers for Disease Control & Prevention, 187 Osongsaengmyeong 2-ro, Osong-eup, Heungduk-gu, Cheongju-si, Chungbuk, 28159 Republic of Korea; 3 WELLS BIO Inc., 16, Magokjungang 8-ro 1-gil, Gangseo-gu, Seoul, 07795 Republic of Korea

**Keywords:** Glucose-6-phosphate dehydrogenase deficiency, Prevalence, Single nucleotide polymorphism, Primaquine

## Abstract

**Background:**

Glucose-6-phosphate dehydrogenase (G6PD) deficiency is the most prevalent inborn disorder. This X-chromosome-linked recessive disease affects more than 400 million people globally, and is associated with haemolytic anaemia after medication with the anti-latent malaria drug, primaquine. To prevent malaria, the Republic of Korea (ROK) Army administers malaria chemoprophylaxis. Due to the previously low G6PD deficiency prevalence in the ROK, prior to primaquine administration, testing for G6PD deficiency was not mandatory. In this study, to evaluate the risk from malaria chemoprophylaxis in the ROK, G6PD deficiency prevalence was investigated.

**Methods:**

Blood specimens from 1632 soldiers entering training camp for the 3^rd^ Infantry of the ROK Army were collected. CareStart™ Biosensor for G6PD and haemoglobin (Hb) was used to detect G6PD levels. G6PD variants using the DiaPlexC G6PD Genotyping kit (Asian type) and full-length sequencing were examined.

**Results:**

Of 1632 blood specimens tested, none was observed to be G6PD deficient. The median value of all tested samples was 7.582 U/g Hb. An investigation of 170 G6PD DNA variants was analysed and categorized as partially low normal [n = 131, 30–80% (2.27–6.05 U/g Hb) of the median value], high [n = 3, > 150% (> 11.373 U/g Hb) of the median value], or normal [n = 36, 80–150% (6.05–11.373 U/g Hb) of the median value], and none was amplified by the DiaPlexC kit. Five silent mutations (C→T) in 131 partially low normal specimens were found at the 1311th nucleotide position by sequence analysis. Another 8 silent mutations (T93C) were also detected in 131 partially low normal specimens. Thus, it is inferred that these silent mutations could be related to G6PD activity.

**Conclusions:**

This G6PD deficiency prevalence study, conducted among participants from the 3rd Infantry of the ROK Army, provided crucial evidence for the safety of malaria chemoprophylaxis. This study showed that the prevalence of G6PD deficiency among 1632 young soldiers was wholly absent. Although G6PD phenotypic mutations were not detected, many silent mutations (C1311T and T93C) were observed. Thus, it is inferred that malaria chemoprophylaxis is relatively safe against G6PD deficiency-mediated haemolytic anaemia. However, given the number of individuals whose G6PD were at the partially low normal range and the frequent detection of G6PD deficiency-related mutations, consistent monitoring of G6PD deficiency is needed.

## Background

Glucose-6-phosphate dehydrogenase (G6PD) is an X-chromosome-linked enzyme involved in the pentose phosphate pathway, and it plays a pivotal role in defending against oxidative stress by generating NADPH (coenzyme nicotinamide adenine dinucleotide phosphate) [1–3]. G6PD deficiency is the most common enzyme deficiency disease with 400 million patients worldwide [4], and a global prevalence rate that varies from 0.1 to 30% [5, 6]. Previous studies have reported 0.9–3.5% prevalence in Republic of Korea (ROK) [7–9].

Malaria, a mosquito-borne parasitic disease, causes more than 200 million cases every year, resulting in about 700,000 deaths [10, 11]. Gyeonggi Province, in ROK, is endemic for *Plasmodium vivax*, a parasite that causes malaria. Gyeonggi is near the demilitarized zone (DMZ); many military personnel are stationed there and are at high risk of malaria [12]. For many centuries, the Korean peninsula was Vivax malaria-endemic until the late 1970s when ROK declared malaria free. Since its re-emergence in 1993, the incidence of malaria infections has been steadily increasing near DMZ [13]. Since 1997, the ROK Army has carried out chemoprophylaxis with chloroquine and primaquine to prevent malaria. [Regimen: Hydroxychloroquine (300 mg) was administrated weekly, during the malaria transmission period (July–November), and subsequently primaquine (15 mg) was administrated daily for 14 days]. In current, the drug tolerance induced by malaria chemotherapy or chemoprophylaxis (chloroquine and primaquine) was less reported in ROK. Thus, other strategy of drug implementation was not considered. But, due to the risk of primaquine-induced G6PD deficiency-mediated haemolytic anaemia, the consideration for the use of other chemotherapy or chemoprophylaxis such as 8-aminoquinoline is also needed [14].

Unlike other malarial protozoans, *P. vivax* exists as a hypnozoite in the liver during part of its life cycle and infects red blood cells (RBCs) [15]. To prevent malaria spread via relapse, it is essential to destroy latent hypnozoites in the liver [15]. Primaquine, an effective drug for eliminating *P. vivax* hypnozoites in their dormant stage, is administrated to about 100,000 military personnel in domestic malaria-endemic areas [13]; it can be administered prophylactically and therapeutically [13]. Primaquine also causes G6PD deficiency-related haemolytic responses [16–18]. Due to adverse haemolytic effects, the World Health Organization (WHO) and US Centers for Disease Control and Prevention (US-CDC) recommend that G6PD screening tests be performed before primaquine administration, but, because of low prevalence of G6PD, screening is not conducted in ROK.

To analyse G6PD deficiency prevalence and malaria chemoprophylaxis risk among military personnel, G6PD enzymatic activity test against new recruits into the 3^rd^ Infantry was performed and genetic analyses was also conducted on samples with activity levels 30–80% or > 150% of the median G6PD level.

As previously mentioned, G6PD deficiency screening has not been implemented in ROK due to low prevalence [7–9]. However, since 1984, G6PD-deficient clinical patients have been increasingly reported in ROK [19–21], and G6PD deficiency-mediated hemolytic anemia patients (G-6-PDH 0.3 U/gHb in 2012 and G-6-PDH 2.6 U/gHb in 2015) were identified in the ROK Army, after primaquine uptake without G6PD screening. Thus, there is a need for periodic monitoring of G6PD deficiency prevalence.

## Methods

### Ethics statement and sample preparation

This study was approved by the ethics committee of the Armed Forces Medical Command (AFMC) of the ROK (Approval No. AFMC-17-IRB-023). Written informed consent was obtained from each participant to collect a 5-ml blood sample. Using educational materials, study objectives and activities were explained to all soldiers who were admitted to recruitment training camps in Paju and Yangju, which are malaria-endemic areas where the military administers malaria chemoprophylaxis. Afterwards, a written study explanation and a research agreement form were distributed to obtain participant consent.

**Fig. 1:**
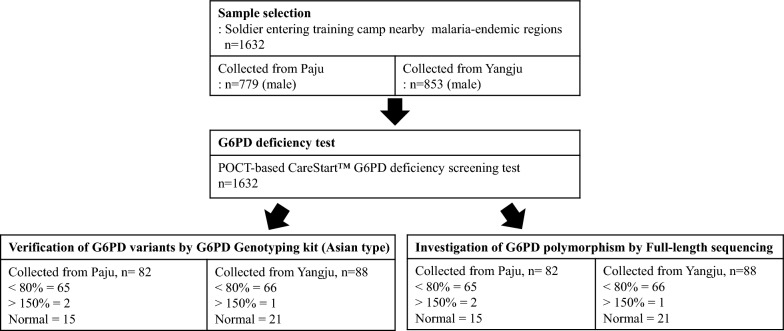
Schematic diagram of sample selection for enzymatic and genetic evaluation of G6PD deficiency. In accordance with IRB-approved (AFMC-17-IRB-023) protocol, blood samples were collected from soldiers who agreed to participate in the study. All 1632 blood samples were collected in two cities, Yangju (n = 853) and Paju (n = 779) and were screened using the CareStart G6PD and Hb POC test. Based on screened results, 134 samples that were below (30–80%; n = 131) and above (> 150%; n = 3) the G6PD median value underwent genetic analysis using a G6PD genotyping kit and full-length sequencing. Thirty-six normal-range G6PD samples were also tested. Paju recruitment training camp: n = 779/1007, consent rate = 77%; Yangju recruitment training camp: n = 853/1011, consent rate = 84%

This study was conducted at the Department of Infectious Disease Research of the Armed Forces Medical Research Institute from April 2017 to January 2018. EDTA-preserved venous blood samples were obtained from 1632 soldier participants who resided in the endemic area (Paju recruitment training camp: n = 779/1007, consent rate = 77%; Yangju recruitment training camp: n = 853/1011, consent rate = 84%; Fig. 1).

### Determination of G6PD deficiency

Point-of-care (POC)-based G6PD and haemoglobin (Hb) testing by venipuncture was performed with CareStart™ Biosensor 1 (Cat No: BBA-E00182; AccessBio, New Jersey, USA) according to the manufacturer’s instructions. Two blood samples, both 10 μl volume, were collected: one was transferred to an Hb strip and the other was spotted onto parafilm and then transferred to a G6PD strip. After 5 min, the CareStart™ Biosensor 1 kit produced estimates of Hb (g/dl) and G6PD (U/dl) concentrations. All samples were tested in duplicate. The artificial blood of G6PD is provided by Biosensor 1 manufacturer (AccessBio, New Jersey, USA). To validate the reliability G6PD activity was evaluated by Glucose-6-Phosphate Dehydrogenase Reagent set (Pointe Scientific, Canton, USA) as reference test. For the reference test, 10 μl of blood was added to 1 ml of R1 reagent in a labelled cuvette, and mixed thoroughly to suspend erythrocytes; then mixture was incubated for 5–10 min at room temperature. After adding 2 ml of R2 reagent to the incubated sample, the cuvette was placed in a 37 °C-water bath for 5 min, and then measured absorbance (A1) at 340 nm. Next, absorbance (A2) at 340 nm after another 5-min incubation was checked. Using these absorbance values, ΔA per min as [(A2-A1)/5] was calculated. By assigning measurements to the following formula [G6PD (U/g Hb) = △A per min × [(100 × 3.01)/(0.01 × 6.22 × Hb (g/dl))] × TCF, G6PD in U/g Hb was calculated. [100 = factor to convert activity 100 ml, 3.01 = total reaction volume (ml), 0.01 = sample volume (ml), 6.22 = millimolar absorptivity of NADPH at 30 nm, Hb (g/dl) = haemoglobin concentration for each specimen, TCF = temperature correction factor (1 at 37 °C)]

### DNA extraction and nested PCR diagnosis

Genomic DNA was extracted from 200 μl of whole blood using the DNeasy Blood and Tissue Kit (Qiagen, Hilden, Germany), according to manufacturer’s instructions. Purified DNA samples were diagnosed using nested polymerase chain reaction (PCR) and the G6PD gene was amplified using specific primers [22]. The first PCR round was performed under the following conditions: 94 °C for 1 min, followed by 38 cycles at 94 °C for 12 s, 65 °C for 30 s, and 68 °C for 6 min, and a final extension at 68 °C for 10 min. The amplification reaction was carried out in 20 μl volume reactions, including primer volumes (1 μl of forward and reverse primer, 5 pmol/μl), 10 μl of PCR master mix (Takara Bio Inc, Shiga, Japan), and 3 μl of DNA template. The amplified products from the first round were subjected to a second PCR round with primers (Fig. 5). The second-round amplification reaction was carried out in 20-μl volume reactions, including primers (1 μl of forward and reverse primer, 5 pmol/μl), 10 μl of PCR master mix (Takara Bio Inc., Shiga, Japan), and 3 μl of DNA template. The second PCR round was performed under the following conditions: 94 °C for 1 min, followed by 35 cycles at 94 °C for 12 s, 62 °C for 25 s, and 72 °C for 3 min, and a final extension at 72 °C for 5 min. Each fragment was analysed with 2 sequencing primers (Fig. 5).

### Analysis of G6PD variants

The genotyping on 170 blood samples representing different ranges of G6PD activity level [n = 131 samples with 30–80% (2.27–6.05 U/g Hb; partially low normal) of the median; n = 3 samples with > 150% (> 11.373 U/g Hb; high) of the median; and n = 36 samples within the 80–150% (6.05–11.373 U/g Hb; normal) of the median] was performed. G6PD variants were detected using the DiaPlexC G6PD Genotyping Kit (Asian type; SolGent, Daejeon, ROK), which specifically screens for the seven representative Asian variants of the G6PD gene via one-step PCR. The variants each produce PCR products of different sizes: Vanua Lava (383 T>C), Mahidol (487 G>A), Coimbra (592 C>T), Viangchan (871 G>A), Union (1360 C>T), Canton (1376 G>T), and Kalping (1388 G>A). PCR mixtures were prepared with 2 μl of genomic DNA (internal control, wild-type control, or mutation control) and 23 μl of master mix (12.5 µl polymerase premix, 2 µl primer premix, and 8.5 µl distilled water). The PCR cycling conditions were as follows: initial denaturation at 95 °C for 15 min, 30 amplification cycles (denaturation at 95 °C for 30 s, annealing at 60 °C for 30 s, elongation at 72 °C for 40 s), and a final extension at 72 °C for 5 min. PCR products were resolved by electrophoresis on 1% agarose gel, stained with SYBR Safe DNA Gel Stain (Thermo Fisher Scientific, Waltham, MA, USA), and visualized using FluorChem FC3 (ProteinSimple, Santa Clara, CA, USA).

### G6PD amplification and G6PD variant sequencing

Of the 170 blood samples genotyped for G6PD, further sequencing on the 134 samples that represented the lower and upper bounds of G6PD activity (n = 131 samples with 30–80% median activity and n = 3 samples with > 150% median activity) and n = 36 samples within the 80–150% median activity was performed. Primer sets used for G6PD amplification and sequencing are listed in Fig. 5. Each fragment was analysed with 2 sequencing primers (Fig. 5). Confirmed sequences were analysed with a wild type sequence using BioEdit v7.0.5 software.

### Statistical analyses

Data are presented as means and standard errors. Statistical analyses were performed using GraphPad Prism version 7 (GraphPad Software, La Jolla, CA, USA). The significance of pairwise comparisons was determined using the Student’s *t* test, and p < 0.05 was considered significant.

## Results

### Schematic diagram of sample selection for enzymatic and genetic evaluation of G6PD deficiency

As shown in Fig. 1, activity-based phenotypic and DNA-based genotypic tests were performed to estimate G6PD deficiency prevalence in the ROK Army. With approval from the AFMC IRB (AFMC-17-IRB-023), a POC-based G6PD activity test by venipuncture using the CareStart G6PD & Hb kit was performed for 1632 newly recruited soldiers at the Paju and Yangju training camps, where malaria is endemic. Genetic sequencing of G6PD was also performed on 170 soldiers’ samples, which showed partially low normal, high, or normal G6PD activity.

### Screening and classification of G6PD activity from 1632 blood samples

**Fig. 2:**
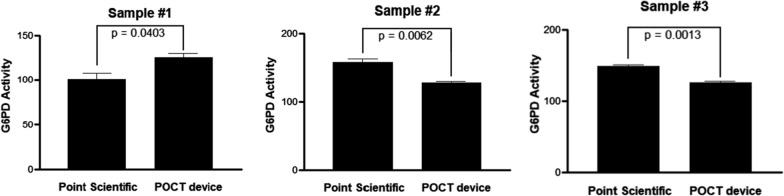
Comparison of two analytical methods for representing G6PD activity: Point Scientific G-6-PDH kit and CareStart G6PD Biosensor. Using two analytical methods (Pointe Scientific G-6-PDH kit and CareStart G6PD Biosensor), G6PD activity from 3 representative samples are presented. Significance was calculated using an unpaired, two-tailed t-test (mean ± SEM; n = 3 for both the Point Scientific and the POCT Analyzer tests)

All 1632 blood samples analysed in this study were collected in two cities, Yangju (n = 853) and Paju (n = 779), and all were screened using the CareStart G6PD and Hb POC test and the Pointe Scientific G-6-PDH kit as a reference test based on quantitative enzyme kinetics. Although the Pointe Scientific kit is commonly used, it is labour-intensive and time-consuming. The CareStart test is rapid and convenient and is a suitable alternative to G6PD enzyme assays. The results from these two methods showed similar activity values within the error range (Fig. 2). Due to the poor correlation by normal G6PD samples comparison, POC-based G6PD testing using artificial bloods of G6PD is also checked (Additional file 1: Table S1).

**Fig. 3:**
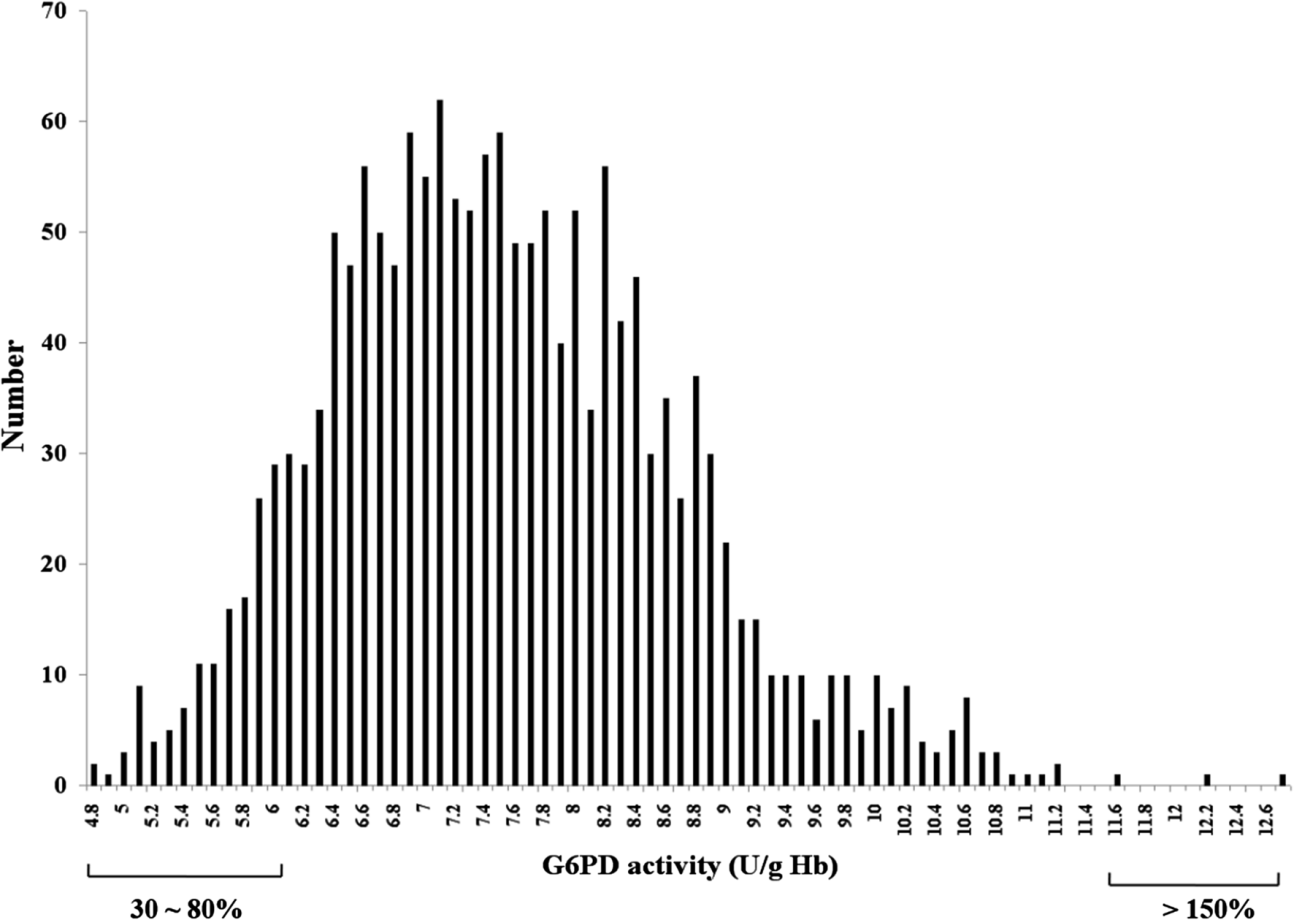
Distribution of G6PD activity. G6PD activity values for all 1632 participants fell within the normal range G6PD values (2.27–11.373 U/gHb), and the median value was 7.582 U/gHb

The standard range for normal G6PD enzyme activity has been defined to be 2.27–11.373 U/gHb (30–150% of median value). All participants were confirmed to have G6PD levels within 30–150% of median activity (Additional file 2: Figure S1). G6PD activity distribution for all samples is shown in Fig. 3. According to the WHO G6PD POC testing guidelines, values < 30% of the median are defined as deficient. Among female patients, 30–80% of the median is classified as intermediate deficiency. Of the 1632 participants, 131 were within 30–80% of median activity (2.27–6.05 U/gHb) and no one had < 30% median activity (< 2.27 U/gHb).

### G6PD-genetic analysis using the G6PD genotyping kit and full-length sequencing

**Fig. 4:**
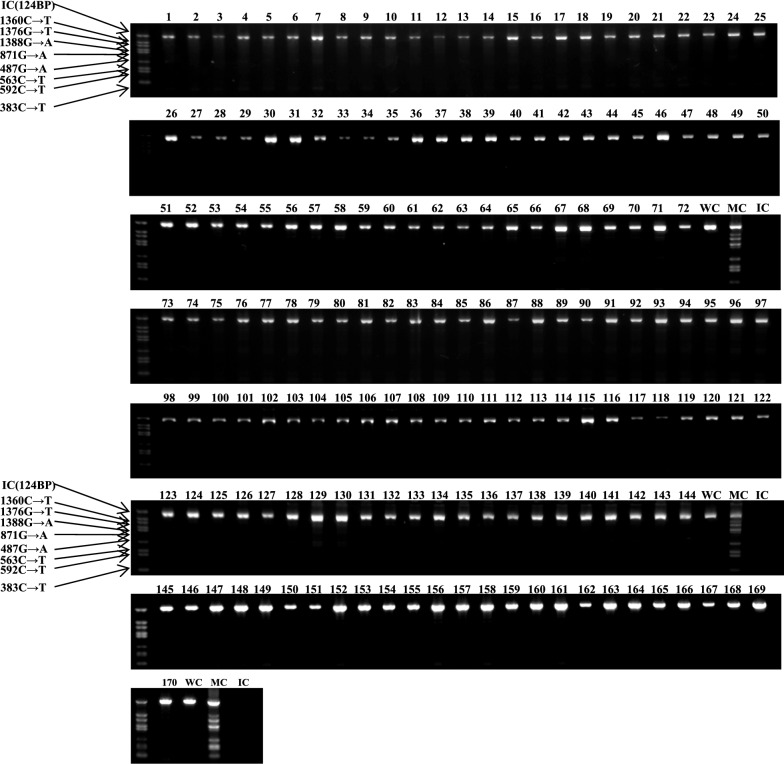
Screening results from seven representative G6PD variants using the DiaPlexC G6PD genotyping Kit (Asian type). To detect 7 different G6PD variants, including Vanua Lava (383 T>C), Mahidol (487 G>A), Coimbra (592 C>T), Viangchan (871 G>A), Union (1360 C>T), Canton (1376 G>T), and Kalping (1388 G>A), 170 blood samples representing different ranges of G6PD activity level [n = 131 samples with partially low normal, 30–80% (2.27–6.05 U/g Hb) of the median; n = 3 samples with high, > 150% (> 11.373 U/g Hb) of the median; and n = 36 samples with normal, 80–150% (6.05–11.373 U/g Hb) of the median] (Among 170 samples, 82 samples were collected from Paju and 88 samples from Yangju) were screened with the one-step PCR method of the DiaPlexC kit. IC: internal control, WC: wild-type control, MC: mutant-type control

**Fig. 5:**
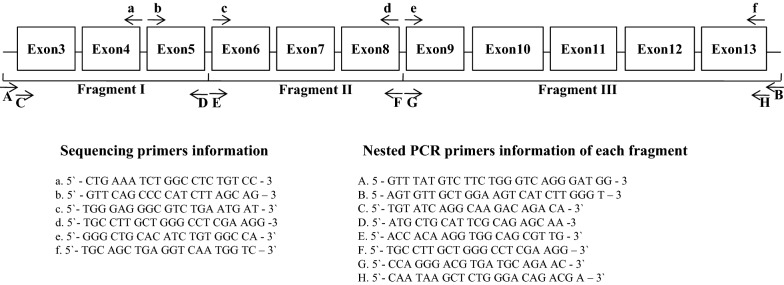
Primer schematic for G6PD gene sequencing. For further SNP evaluation of G6PD (from exon 3 to exon 13), nested PCR and sequencing primers were designed

**Fig. 6:**
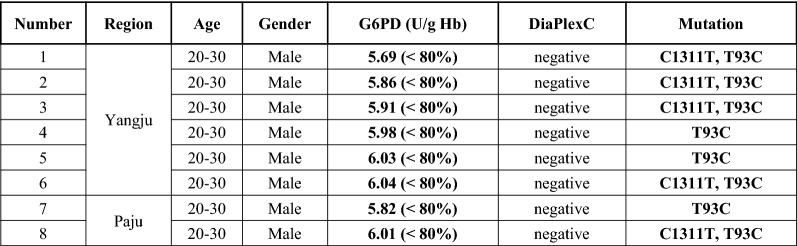
Profiles of eight participants with mutations. Basic information (region, age, gender, and G6PD activity) of participants with a C1311T exon mutation or a T93C intron mutation

Using 170 blood samples representing different ranges of G6PD activity level [n = 131 samples with 30–80% (2.27–6.05 U/g Hb; partially low normal) of the median; n = 3 samples with > 150% (> 11.373 U/g Hb) of the median; and n = 36 samples within the 80–150% (6.05–11.373 U/g Hb) of the median], one-step PCR genotyping, using G6PD Genotyping Kit (Asian type; Solgent), was conducted to detect 7 G6PD variants: Vanua Lava (383 T>C), Mahidol (487 G>A), Coimbra (592 C>T), Viangchan (871 G>A), Union (1360 C>T), Canton (1376 G>T), and Kalping (1388 G>A). As shown in Fig. 4, any bands suspected to contain G6PD variants is not detected. To identify other potential single nucleotide polymorphisms (SNPs), G6PD sequencing analysis, using nested PCR, was performed, as depicted in Fig. 5. This study found several C1311T exon mutations and T93C intron mutations. All mutations are listed in Fig. 6. The C1311T/IVS polymorphism frequency at exon 11 of the G6PD gene is shown as 5/170 or 0.029. The other silent mutation (T93C), at intron 11 is shown as 8/170 or 0.047. All sequencing data from the 131 partially low normal, 36 normal, and 3 high G6PD activity samples are provided in Additional file 3: Figure S2. Previous studies have reported an association between these mutations [23, 24], which is consistent with this study’s finding of a frequency of 5/8 (0.625) for combined mutations of C1311T and T93C. Of the 8 T93C mutations in intron 11, 5 were not linked to C1311T/IVS. Among the 36 normal-range G6PD samples, no mutation was found. So, it is inferred that the C1311T mutation and the T93C intron single mutation could be linked to G6PD activity.

## Discussion

In 1993, malaria re-emerged as an endemic pathogen in regions near the demilitarized zone (DMZ) and, after a rapid increase in the number of malaria patients in 1997, the ROK Army began conducting malaria chemoprophylaxis with chloroquine for malaria clearance in blood and primaquine to prevent latent malaria [13]. When people who are G6PD deficient take primaquine, they can experience drug-mediated haemolytic adverse effects. Consequently, the WHO and the US-CDC recommend conducting G6PD screening before primaquine administration. However, screening is not performed in ROK because of low prevalence of G6PD deficiency compared with the average global prevalence. Blackwell et al. [8] found that G6PD prevalence was 0.9% among 2595 study participants in 1968, and Saha et al. [7] estimated 3.5% G6PD prevalence among 140 participants in 1984. A recent Korea Centers for Disease Control and Prevention (KCDC) investigation found no cases among 1044 participants sampled [9]. However, since 1992, G6PD-deficient clinical cases have been continuously reported at domestic hospitals, possibly as a consequence of increased immigration from Southeast Asia [21, 25], where G6PD deficiency prevalence is relatively high. G6PD deficiency incidence is expected to rise. In 2012 and 2015, primaquine-uptake-induced G6PD deficiency-mediated haemolytic anaemia [G-6-PDH 0.3 U/gHb (2012) and G-6-PDH 2.6 U/gHb (2015)] occurred due to the absence of G6PD deficiency screening. This highlighted the need for periodic monitoring of G6PD deficiency and was a motivation for this study.

On 1632 blood samples from soldiers who were admitted to recruitment training camps in a malaria-endemic region, a POC G6PD activity test using the CareStart G6PD & Hb kit was performed. All samples’ G6PD activities are within 30-150% of median activity. Based on classifications by Beutler et al. [26], samples < 60% and > 150% of the median value were also examined. No participants had < 60% (4.55 U/gHb), but 3 had > 150% (> 11.373 U/gHb) median activity. Although the sample size of this study is relatively small compared with the number of people who receive military-administered malaria chemoprophylaxis, this study indicates that G6PD deficiency prevalence is low enough that the risk of primaquine-induced G6PD deficiency-mediated haemolytic anaemia is relatively rare. However, there is a large number of military personnel at the border, and C1311T/IVS and T93C intron mutations, which are known to be associated with G6PD deficiency, were detected in this study. This finding suggests that consistent monitoring of G6PD deficiency is necessary to prevent adverse drug-reaction of primaquine administration. A previous report found that C1311T/IVS mutations are linked to the Mediterranean (563C>T) and G6PD Viangchan (871G>A) phenotypic mutations [27]. No associations with these mutations were confirmed in this study. However, the combined mutation of C1311T and T93C, and the T93C single intron mutation were only detected in the 30–80% partially low normal activity group, and not in the > 150% of median value group or in the normal group. Thus, these mutations could be linked to G6PD activity level through their presence in the partially low normal group. Each mutation activity value was investigated according to the description in Fig. 6. All activity values of mutation fell around the upper border of the partially low normal range. Thus, it could be inferred that this mutation is somewhat related to G6PD activity.

Although any G6PD-deficient participants with activity < 30% (< 2.27 U/g Hb) of the median value is not identified, the periodic surveillance for G6PD deficiency is still arguing, due to the relatively high incidence of deficiency-related mutations (C1311T and T93C). Considering the recent case reports of G6PD deficiency-mediated haemolytic anaemia in this malaria-endemic area and the implementation of military malaria chemoprophylaxis, ultimately, screening for deficiency prior to primaquine administration is essential.

## Conclusions

This G6PD deficiency prevalence study showed that G6PD deficiency prevalence among 1632 young soldiers was wholly absent. Although G6PD phenotypic mutations were not detected, 8 silent mutations (C1311T or T93C) for the first time in ROK were identified. Thus, it could be concluded that malaria chemoprophylaxis is relatively safe against G6PD deficiency-mediated haemolytic anaemia. However, the frequent detection of G6PD deficiency-related mutations (C1311T or T93C) in this study is observed. Thus, the consistent monitoring of G6PD deficiency is required in malaria chemoprophylaxis-executed area of ROK.

## Supplementary information

**Additional file 1.** POC-based G6PD activity (U/dL) testing using artificial blood of G6PD.

**Additional file 2.** G6PD activity (U/gHb) of all participants.

**Additional file 3.** Sequencing data of 131 partially low normal, 36 normal, and 3 high G6PD activity samples.

## Data Availability

All data pertaining to this study are within the manuscript and the supporting files.

## Abbreviations

G6PD: Glucose-6-phosphate dehydrogenase deficiency
ROK: Republic of Korea
NADPH: Coenzyme nicotinamide adenine dinucleotide phosphate
DMZ: Demilitarized zone
RBCs: Red blood cells
WHO: World Health Organization
US-CDC: United States Centers for Disease Control and Prevention
AFMC: Armed Forces Medical Command
POC: Point-of-care
PCR: Polymerase chain reaction
KCDC: Korea Centers for Disease Control and Prevention
