# Supplementary material for: Investigation of glucose-6-phosphate dehydrogenase (G6PD) deficiency prevalence in a Plasmodium vivax-endemic area in the Republic of Korea (ROK)
Source: Malar J. 2020 Sep 1;19:317. doi: 10.1186/s12936-020-03393-4 (PMC7465311; doi:10.1186/s12936-020-03393-4)
Supplement: Supplementary file 1 — Additional file 1. POC-based G6PD activity (U/dL) testing using artificial blood of G6PD. [file 12936_2020_3393_MOESM1_ESM.ppt]

## Slide 1
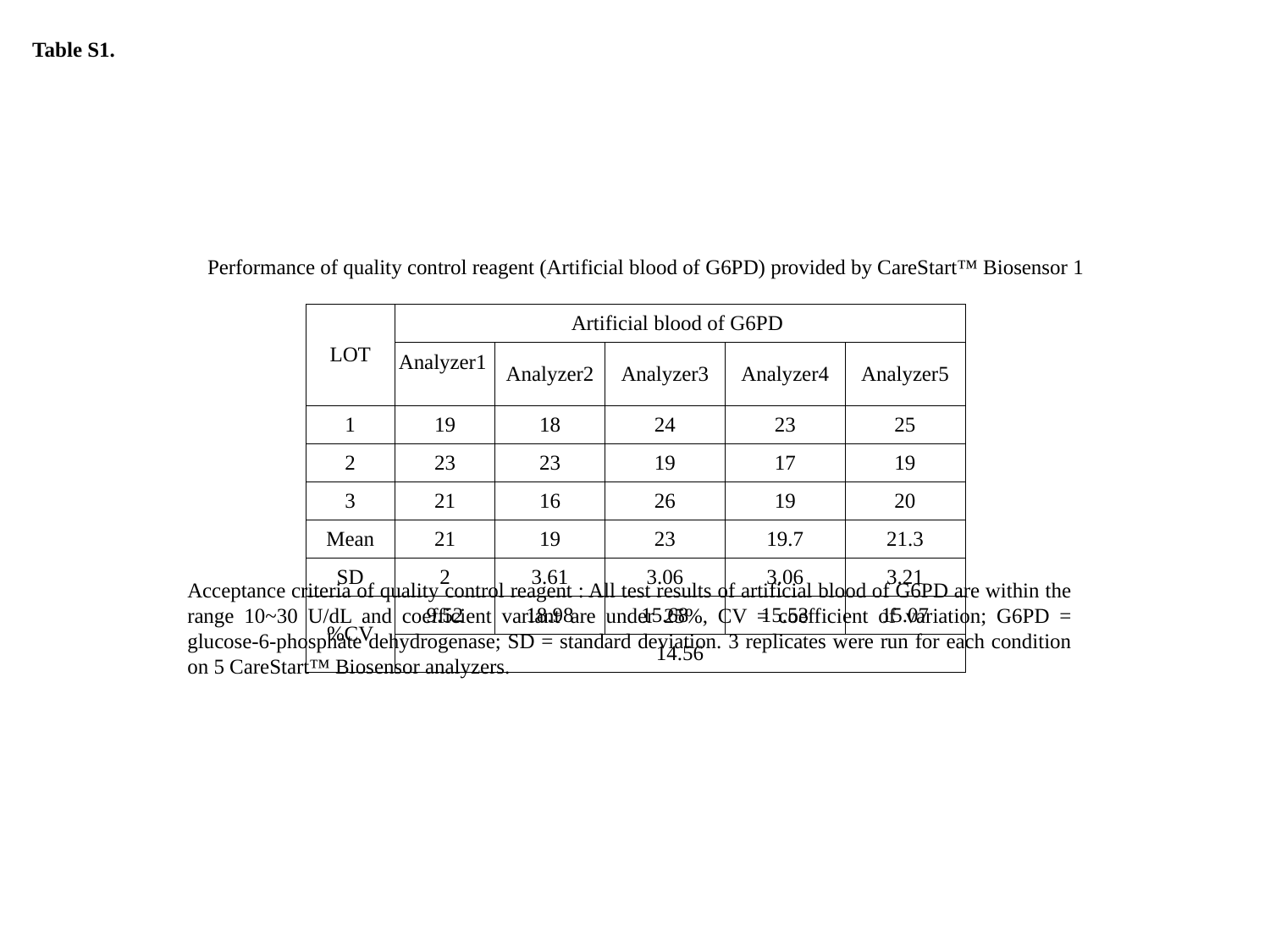

Table S1.
Performance of quality control reagent (Artificial blood of G6PD) provided by CareStart™ Biosensor 1
| LOT | Artificial blood of G6PD | | | | |
| --- | --- | --- | --- | --- | --- |
| | Analyzer1 | Analyzer2 | Analyzer3 | Analyzer4 | Analyzer5 |
| 1 | 19 | 18 | 24 | 23 | 25 |
| 2 | 23 | 23 | 19 | 17 | 19 |
| 3 | 21 | 16 | 26 | 19 | 20 |
| Mean | 21 | 19 | 23 | 19.7 | 21.3 |
| SD | 2 | 3.61 | 3.06 | 3.06 | 3.21 |
| %CV | 9.52 | 18.98 | 15.68 | 15.53 | 15.07 |
| | 14.56 | | | | |
Acceptance criteria of quality control reagent : All test results of artificial blood of G6PD are within the range 10~30 U/dL and coefficient variant are under 25%, CV = coefficient of variation; G6PD = glucose-6-phosphate dehydrogenase; SD = standard deviation. 3 replicates were run for each condition on 5 CareStart™ Biosensor analyzers.
