# Supplementary material for: Investigation of glucose-6-phosphate dehydrogenase (G6PD) deficiency prevalence in a Plasmodium vivax-endemic area in the Republic of Korea (ROK)
Source: Malar J. 2020 Sep 1;19:317. doi: 10.1186/s12936-020-03393-4 (PMC7465311; doi:10.1186/s12936-020-03393-4)

Exon 3

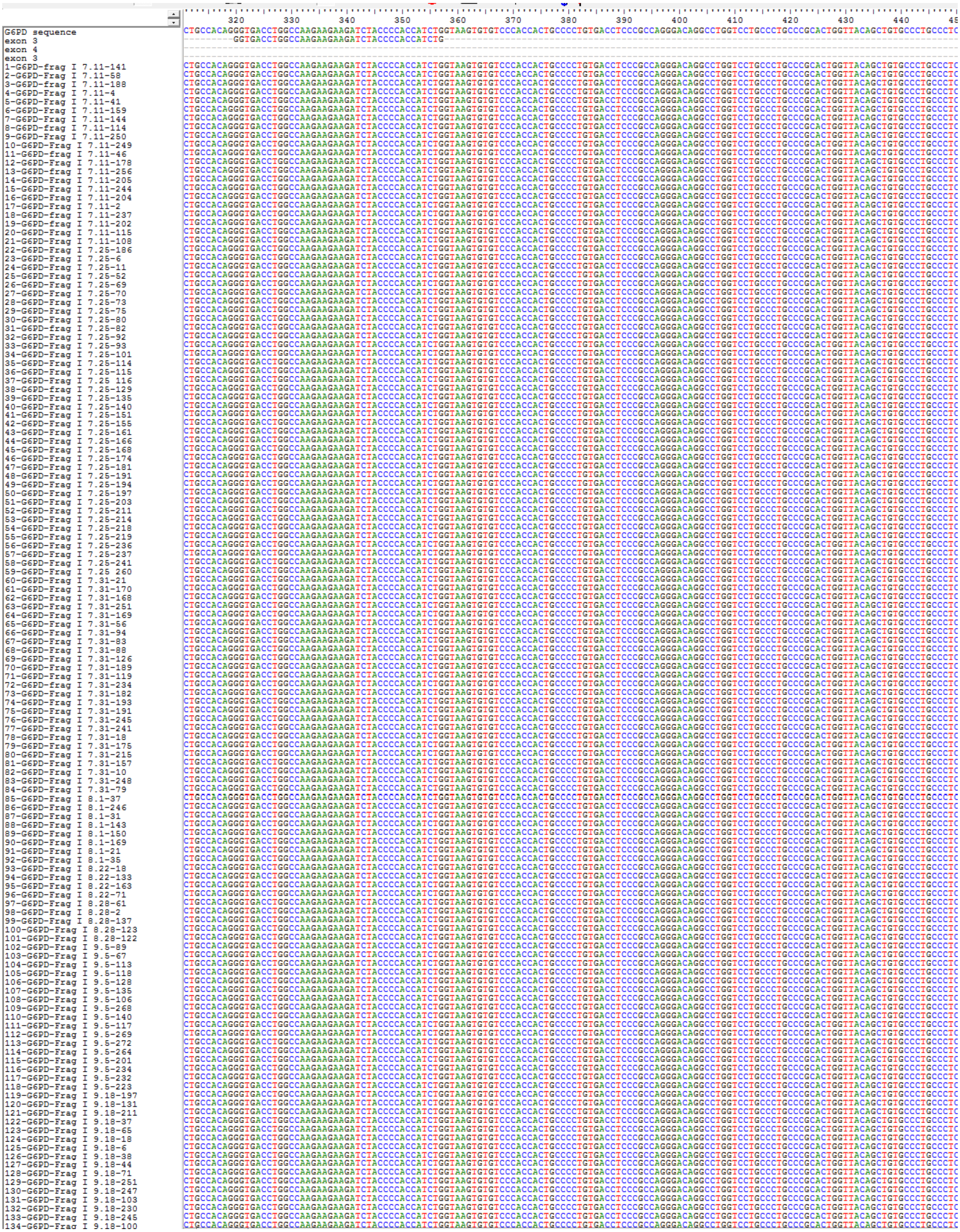
320 330 340 350 360 370 380 390 400 410 420 430 440
G6PD sequence
exon 3
exon 4
exon 3

Exon 4

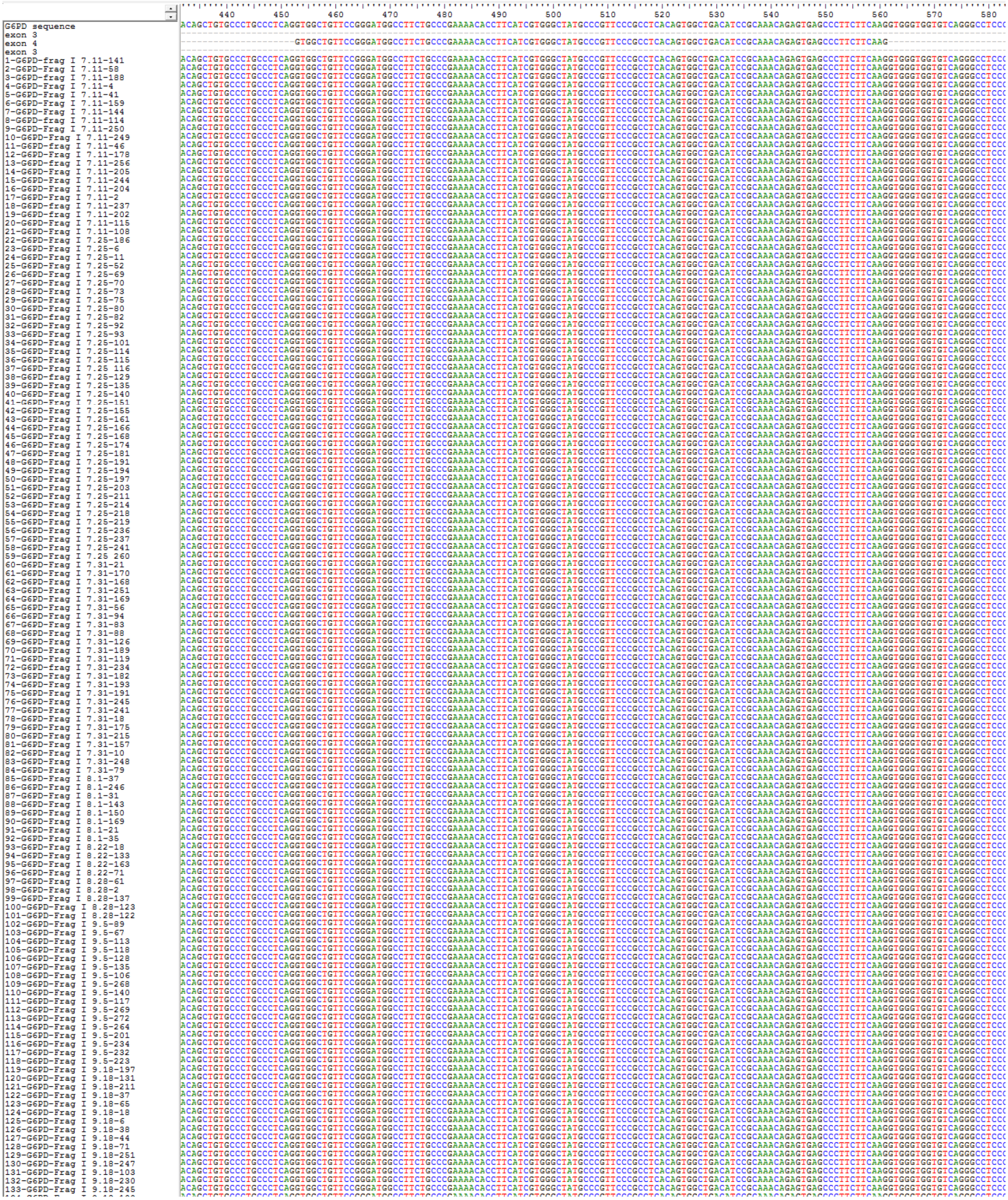

Exon 5

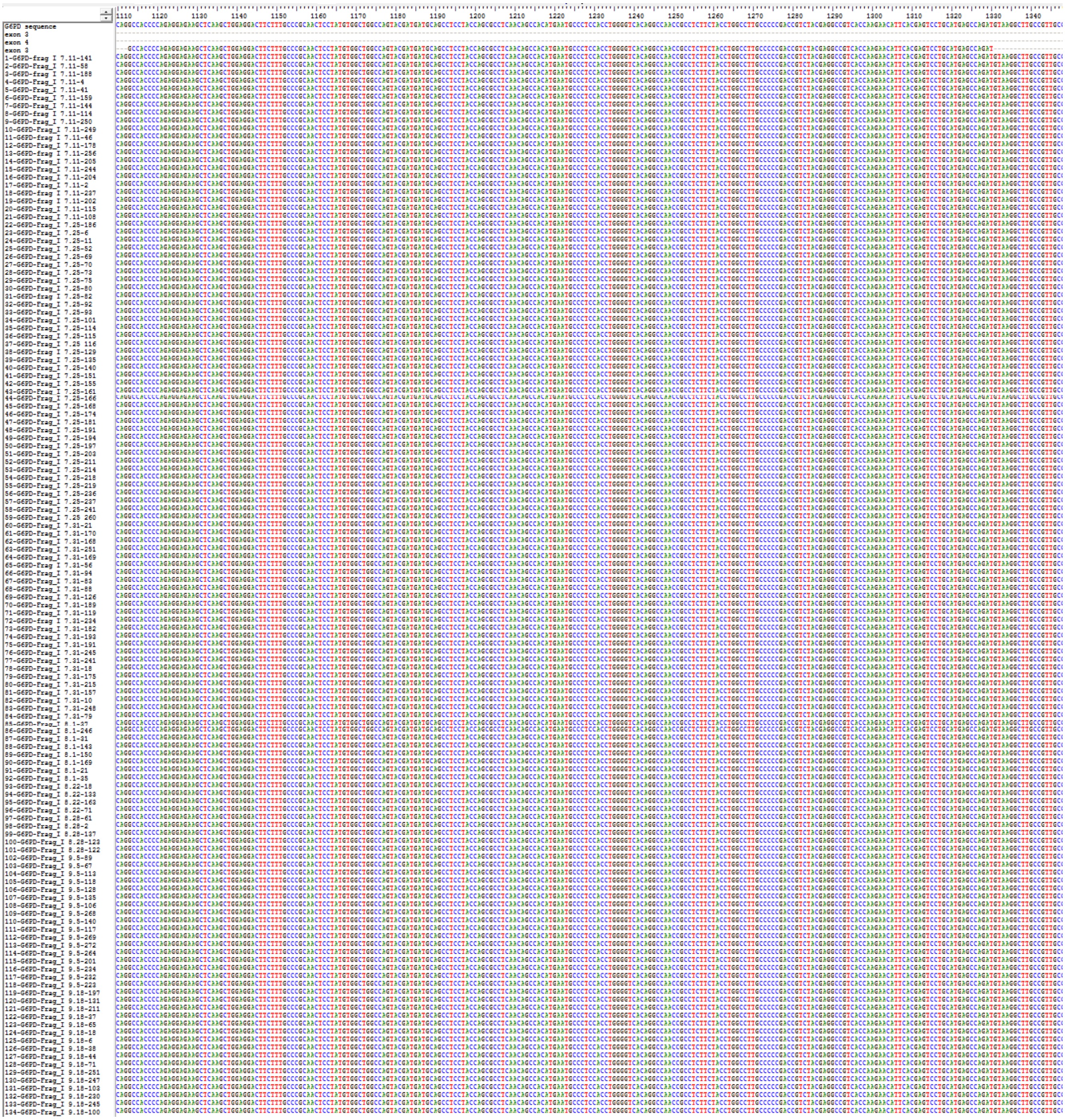

Exon 6

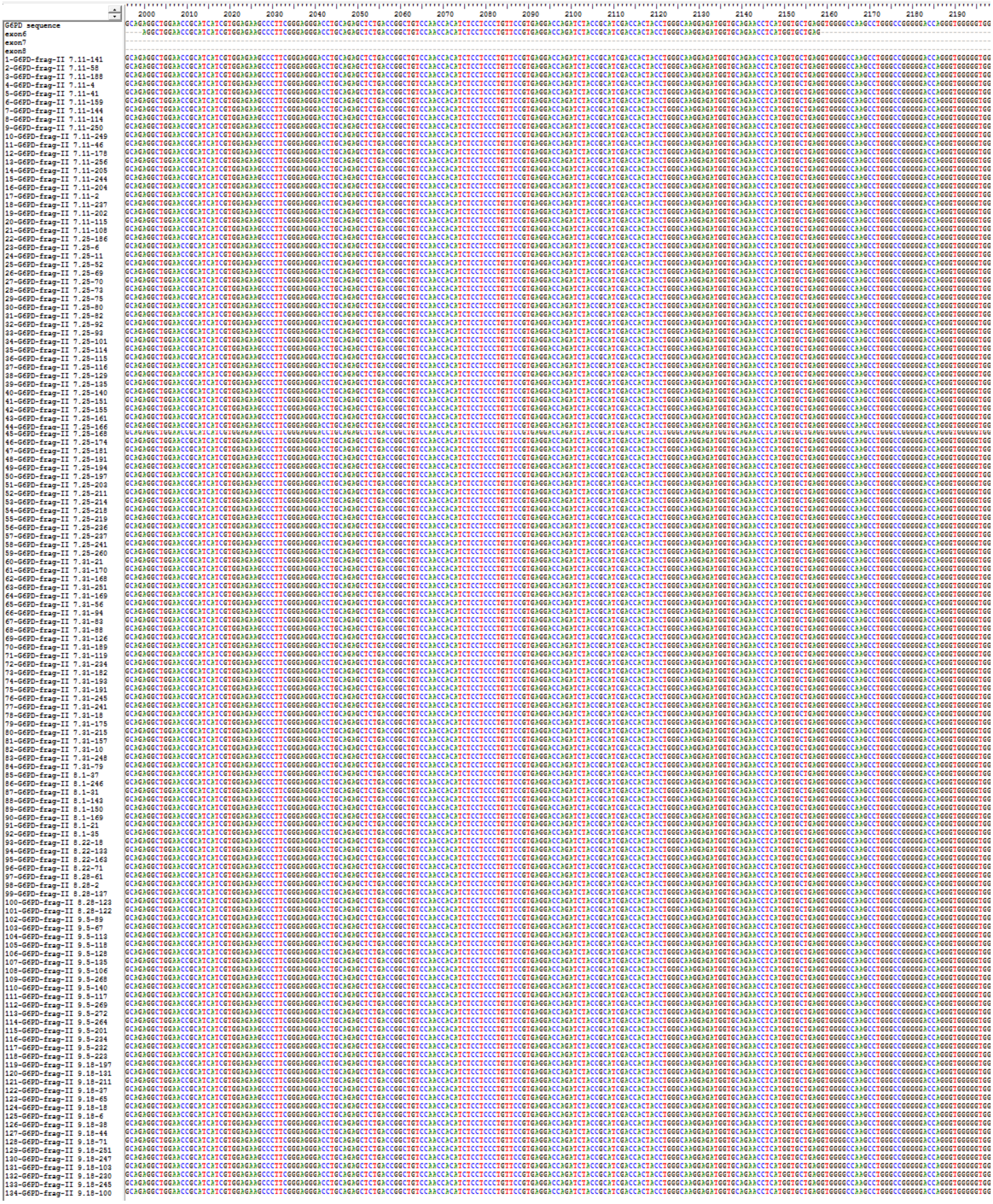

Exon 7

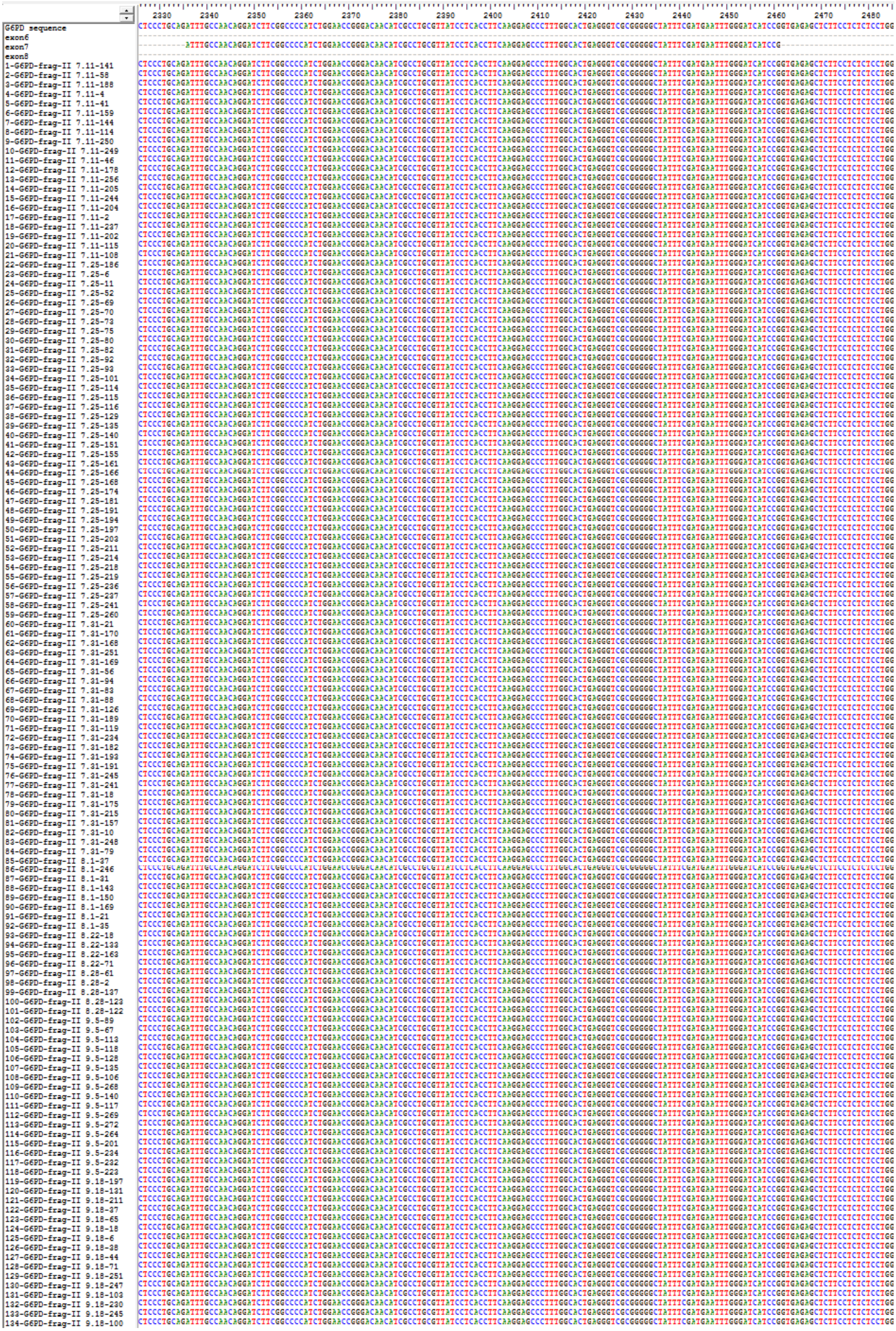
2330
2340
2350
2360
2370
2380
2390
2400
2410
2420
2430
2440
2450
2460
2470
2480
G6PD sequence
exon6
exon7
exon8

Exon 8

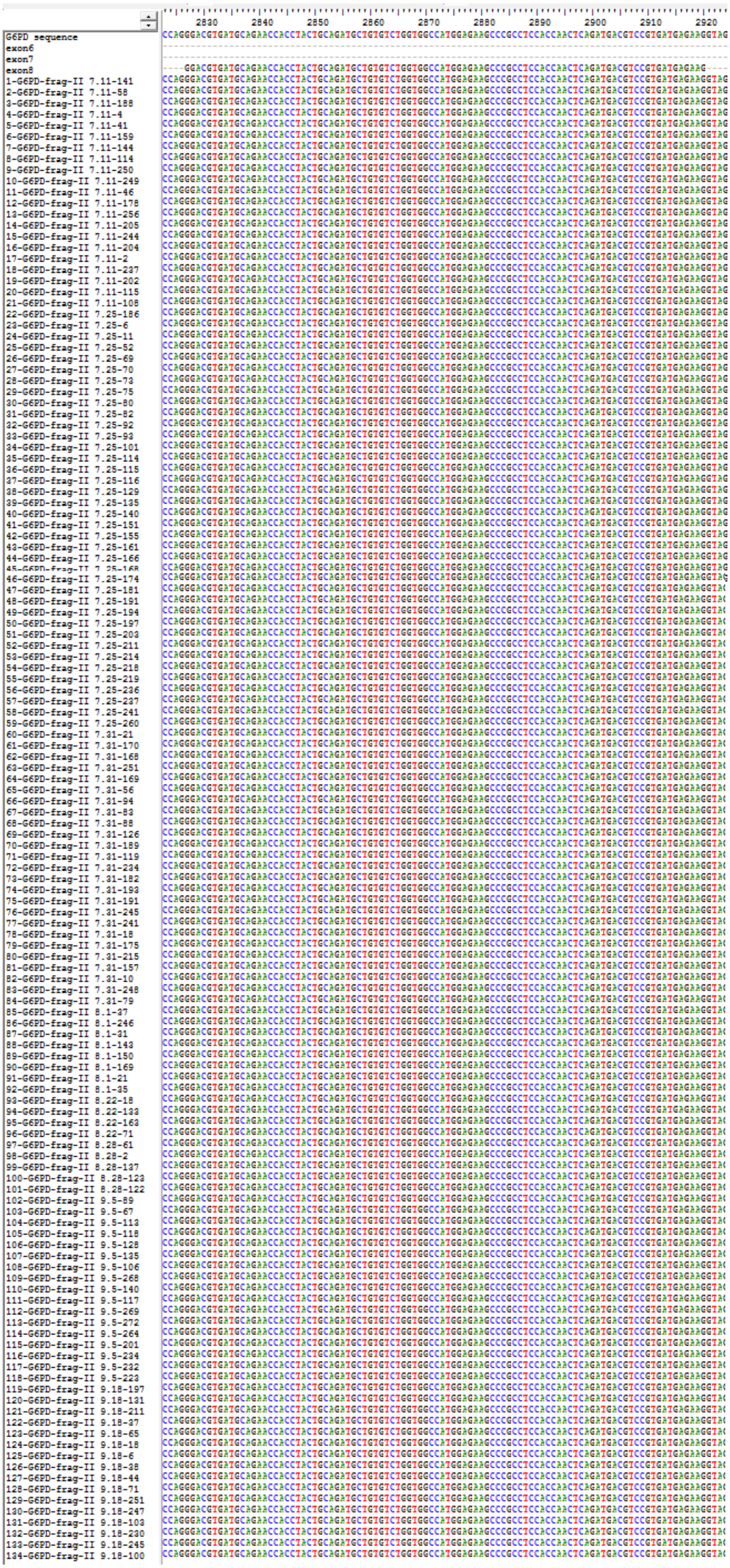
2830 2840 2850 2860 2870 2880 2890 2900 2910 2920
G6PD sequence
exon6
exon7
exon8

Exon 9

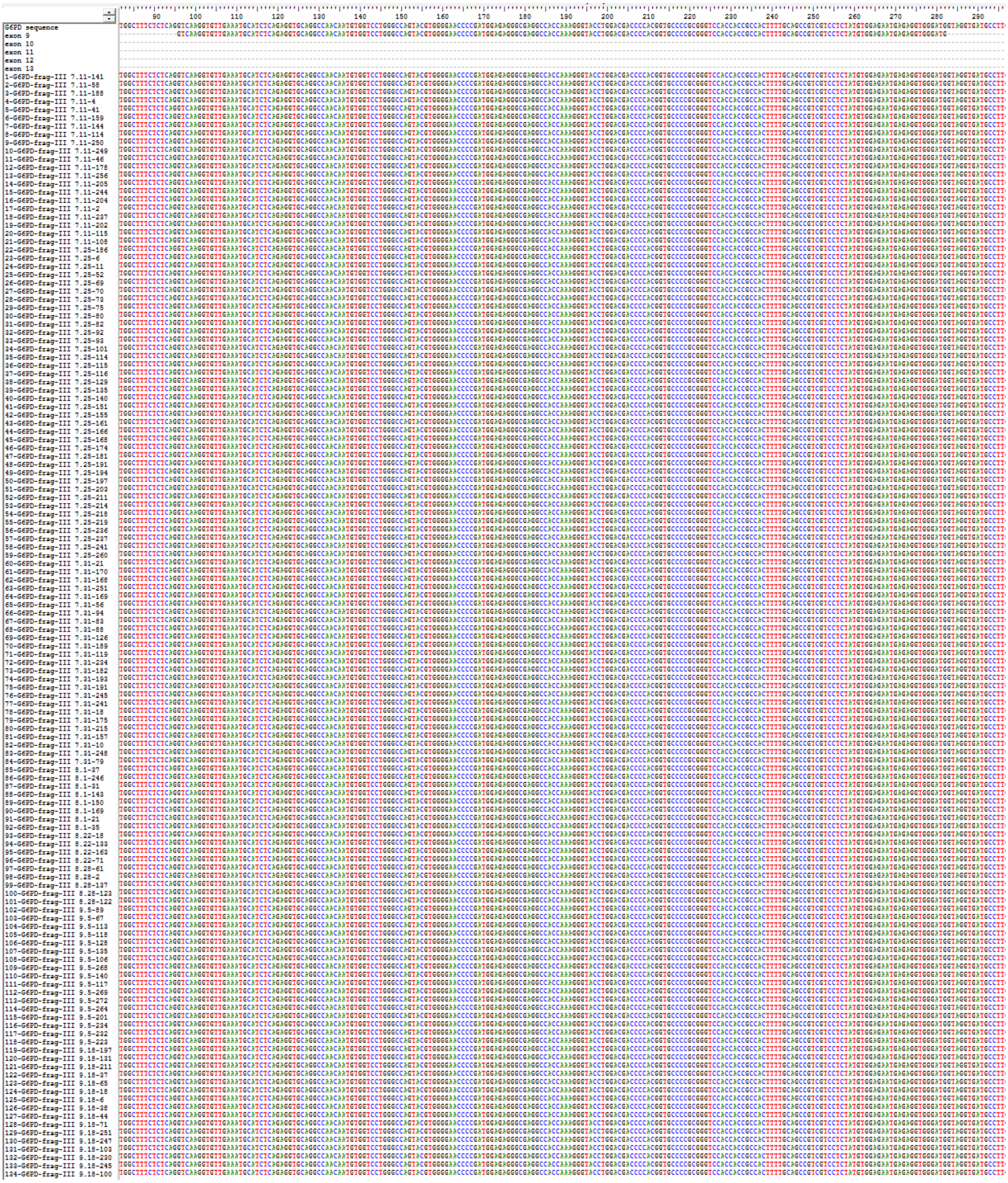

Exon 10

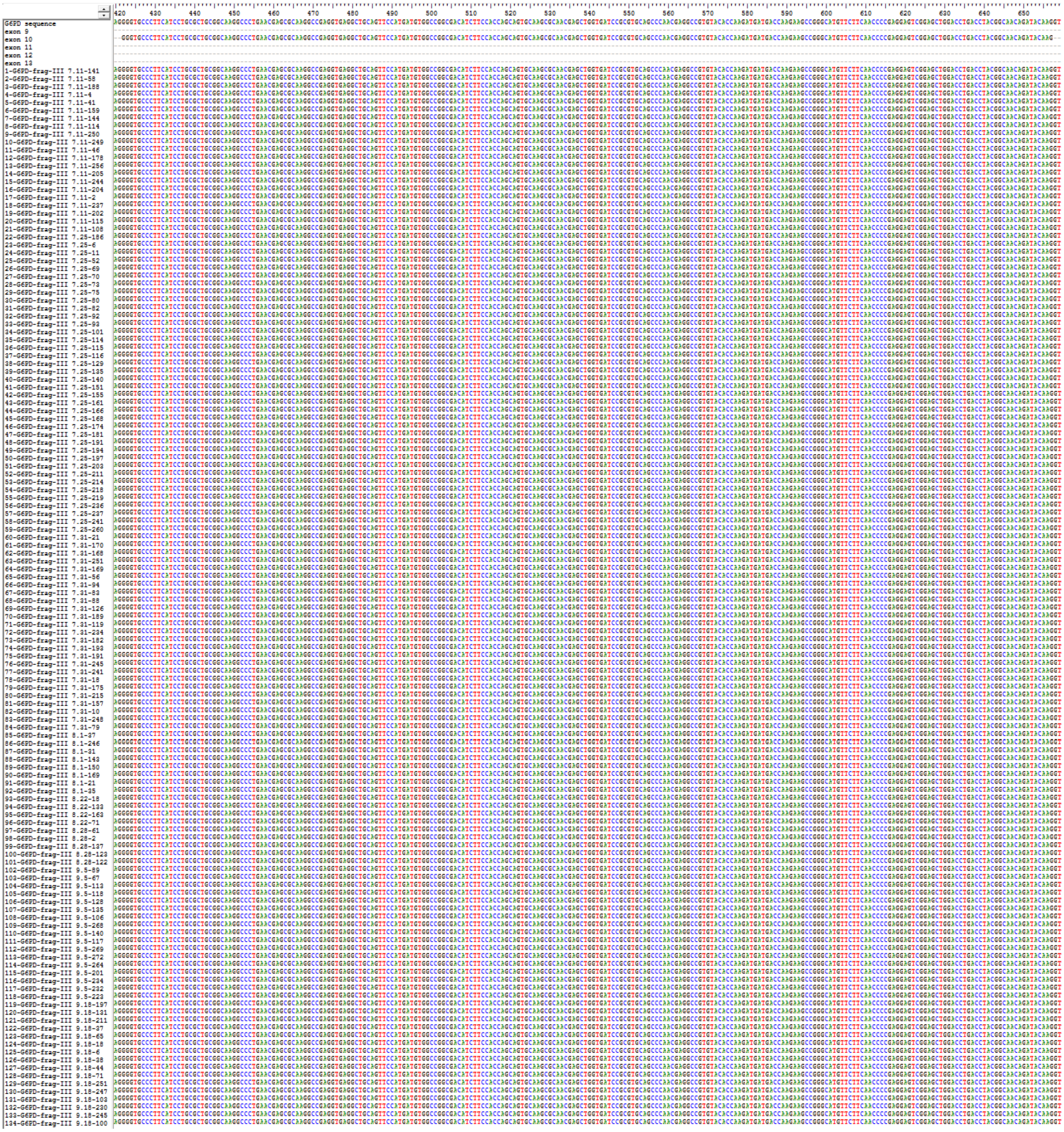

Exon 11

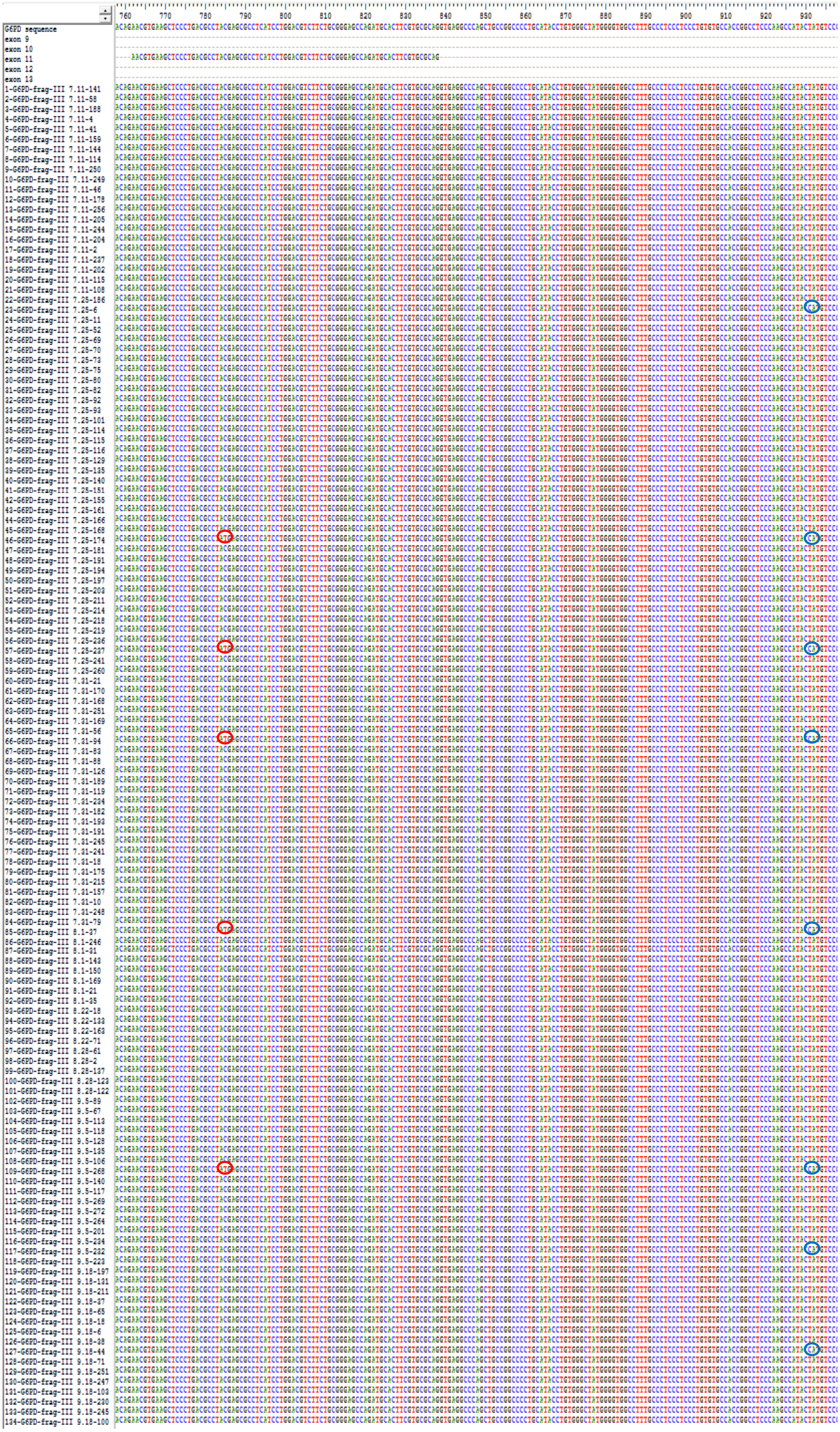

Red = Exon 11 mutation (C->T)
Blue = Intron mutation (T->C)

Exon 12

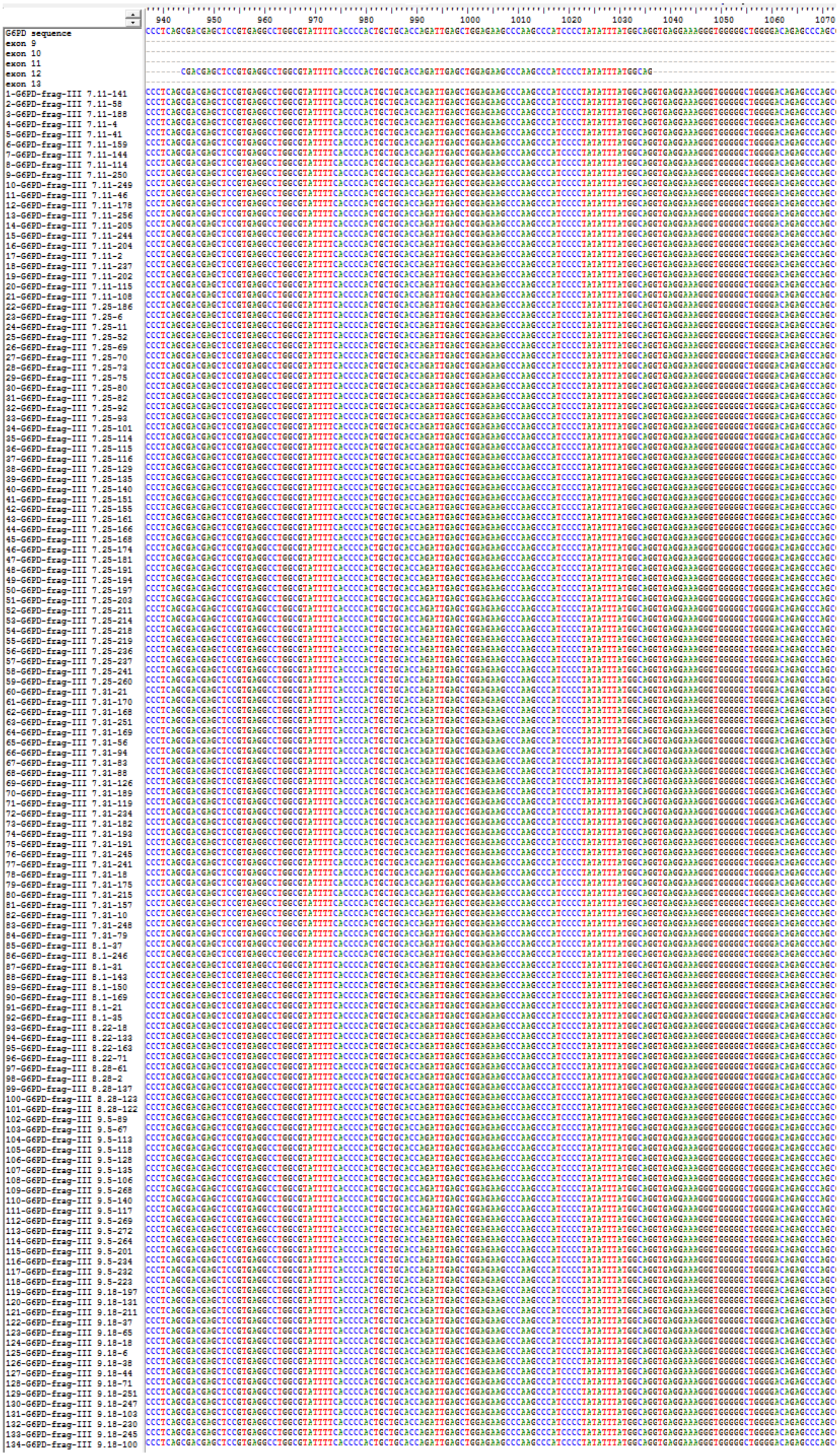

Exon 13

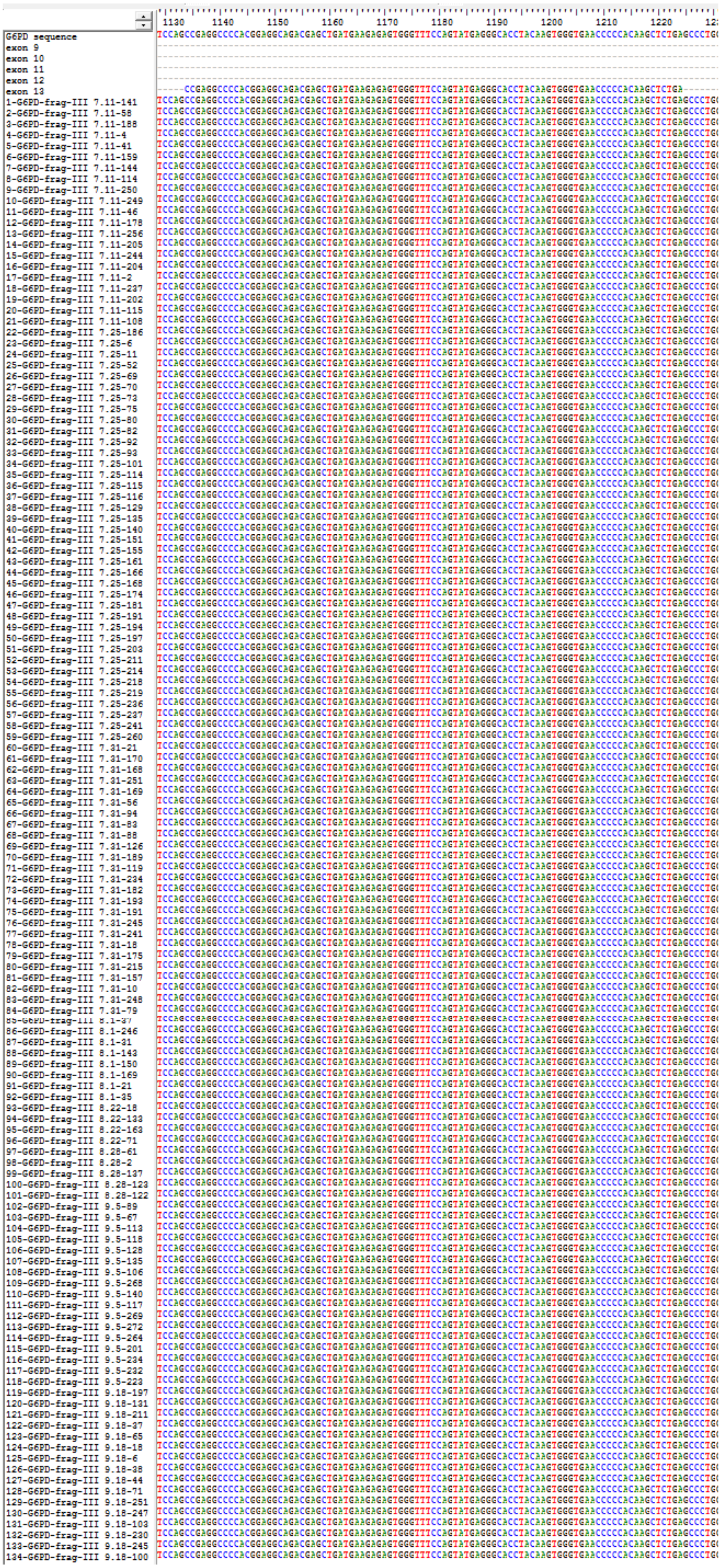

Supplement: Supplementary file 3 — Additional file 3. Sequencing data of 131 partially low normal, 36 normal, and 3 high G6PD activity samples. [file 12936_2020_3393_MOESM3_ESM.pdf]
